# Antagonistic effect of helpers on breeding male and female survival in a cooperatively breeding bird

**DOI:** 10.1111/1365-2656.12377

**Published:** 2015-04-30

**Authors:** Matthieu Paquet, Claire Doutrelant, Ben J Hatchwell, Claire N Spottiswoode, Rita Covas

**Affiliations:** 1 CEFE-CNRS1919 Route de Mende, 34293, Montpellier, France; 2 Percy FitzPatrick Institute, DST-NRF Centre of Excellence, University of Cape TownCape Town, South Africa; 3 Department of Animal and Plant Sciences, University of SheffieldWestern Bank, Sheffield, UK; 4 Department of Zoology, University of CambridgeDowning Street, Cambridge, CB2 3EJ, UK; 5 CIBIO, Research Centre in Biodiversity and Genetic ResourcesCampus Agrário de Vairão, Rua Padre Armando Quintas, 4485-661, Vairão, Portugal; 6 Biology Department, Science Faculty, University of PortoPorto, Portugal

**Keywords:** cooperative breeding, family conflicts, investment, life-history strategies, sex-specific selection

## Abstract

**1.** Cooperatively breeding species are typically long lived and hence, according to theory, are expected to maximize their lifetime reproductive success through maximizing survival. Under these circumstances, the presence of helpers could be used to lighten the effort of current reproduction for parents to achieve higher survival.

**2.** In addition, individuals of different sexes and ages may follow different strategies, but whether male and female breeders and individuals of different ages benefit differently from the presence of helpers has often been overlooked. Moreover, only one study that investigated the relationship between parental survival and the presence of helpers used capture–mark–recapture analyses (CMR). These methods are important since they allow us to account for the non-detection of individuals that are alive in the population but not detected, and thus, the effects on survival and recapture probability to be disentangled.

**3.** Here, we used multi-event CMR methods to investigate whether the number of helpers was associated with an increase in survival probability for male and female breeders of different ages in the sociable weaver *Philetairus socius*. In this species, both sexes reduce their feeding rate in the presence of helpers. We therefore predicted that the presence of helpers should increase the breeders' survival in both sexes, especially early in life when individuals potentially have more future breeding opportunities. In addition, sociable weaver females reduce their investment in eggs in the presence of helpers, so we predicted a stronger effect of helpers on female than male survival.

**4.** As expected we found that females had a higher survival probability when breeding with more helpers. Unexpectedly, however, male survival probability decreased with increasing number of helpers. This antagonistic effect diminished as the breeders grew older.

**5.** These results illustrate the complexity of fitness costs and benefits underlying cooperative behaviours and how these may vary with the individuals' sex and age. They also highlight the need for further studies on the sex-specific effects of helpers on survival.

## Introduction

Cooperative breeding describes the situation where supernumerary sexually mature individuals, named helpers, assist in raising the offspring of others by bringing additional food to the young. It is widespread across animals, both vertebrate and invertebrate (Jennions & Macdonald 1994; Taborsky 1994; Choe & Crespi 1997; Cockburn 1998; Dickinson & Hatchwell 2004). While helping may provide direct benefits to helpers such as a higher future breeding success (Clutton-Brock *et al*. 2002; Richardson, Burke & Komdeur 2002), helpers are often closely related to the parents (Griffin & West 2003) and hence gain indirect genetic benefits by increasing the fitness of these close relatives (Hamilton 1964). This can occur by increasing parents' annual reproductive success and/or their survival (Cockburn 1998; Hatchwell 1999; Khan & Walters 2002; Kingma *et al*. 2010).

Helpers' effects on breeders' survival have been relatively neglected compared to their effect on reproductive success. Cooperatively breeding species are generally long lived (Arnold & Owens 1998) and often live in relatively unpredictable environments (Rubenstein & Lovette 2007). Hence, they are typically predicted to maximize their lifetime reproductive success through maximizing survival, because a small increment in survival probability is likely to result in a considerably higher increase in fitness than a small increase in current reproductive output at the expense of survival (Clutton-Brock 1988; Wilbur & Rudolf 2006). This life-history strategy, coupled with the difficulty of measuring small differences in survival in natural populations, could explain why several studies have failed to find a positive effect of helpers on reproductive success (as found in 12 bird species reviewed in Kingma *et al*. 2010).

Helpers may be beneficial for parental survival because the additional food they provide to the chicks may allow parents to save energy by reducing their own feeding rate and hence their investment in the current brood, if helpers compensate or even overcompensate for this reduction (Hatchwell 1999; Russell *et al*. 2007; Canestrari, Marcos & Baglione 2011). This strategy to increase breeders' survival, termed ‘load-lightening’ (Crick 1992), is especially likely when the probability of future breeding is high, which can arise from high survival probability as well as from a high probability of maintaining breeder status (Russell & Lummaa 2009). A positive effect of helpers on survival is particularly expected early in life since the cost of reproduction is higher due to inexperience (Magrath 2001; Orell & Belda 2002; Krüger 2005; Hawn, Radford & du Plessis 2007). Therefore, the presence of helpers should be especially beneficial in reducing reproductive costs. In addition, the probability of future breeding events is higher (Charlesworth 1994), such that breeders have more to gain from reducing their own contributions to provisioning.

The effect of helpers on breeders' survival may also vary between sexes. For example, in long-tailed tits *Aegithalos caudatus* breeding males reduce their food provisioning in the presence of helpers more than do females, and males but not females are more likely to survive when helped in rearing large broods (Meade *et al*. 2010). Additionally, a comparative study found that an improvement to male (but not female) breeders' survival in the presence of helpers was associated with increased pair fidelity, which might be due to the fact that males adjust their investment depending on their relatedness to the brood (Kingma *et al*. 2010). Finally, several recent studies have shown that, in some species, females reduce their investment in eggs when assisted by helpers (Russell *et al*. 2007; Taborsky, Skubic & Bruintjes 2007; Canestrari, Marcos & Baglione 2011; Santos & Macedo 2011; Paquet *et al*. 2013). Hence, in these species, a stronger effect of helpers might be expected on female survival compared to male survival.

Studies of helpers' effect on survival using capture–mark–recapture (CMR) analyses are extremely rare (but see McGowan, Hatchwell & Woodburn 2003). CMR is the only method currently available to account for the non-detection of individuals by estimating and taking into account recapture probability. Under some circumstances, these methods are essential to model the probability that individuals are present but not detected because failure to do so may result in inappropriate conclusions (Gimenez *et al*. 2008).

Here, we test the hypothesis that helpers increase parental survival in a colonial cooperatively breeding passerine, the sociable weaver *Philetairus socius*. Sociable weavers are socially and genetically monogamous at our study site (Covas *et al*. 2006), and both breeders incubate the eggs and feed the nestlings. Males feed at higher rates than females (Doutrelant & Covas 2007), but both breeding males and females reduce their provisioning effort at a similar rate when helped (Covas, du Plessis & Doutrelant 2008). We can thus expect a positive effect of helpers on both male and female survival. In addition, we expect these effects to be particularly marked early in life when individuals may face greater costs of reproduction due to inexperience and have potentially higher future breeding opportunities. Additionally, sociable weaver females lay lighter eggs when assisted by helpers (Paquet *et al*. 2013). Therefore, we predict a greater positive effect of the presence of helpers on female than on male survival probability.

## Materials and Methods

### Study species

The sociable weaver is a passerine endemic to the semi-arid acacia savannas of southern Africa (Maclean 1973a; Mendelsohn & Anderson 1997). Sociable weavers build massive communal nests containing a variable number of independent nest chambers that are used for breeding and roosting. They are facultative cooperative breeders, breeding in pairs or with up to five helpers (mean group size = 3·15 birds, however, the proportion of birds breeding in groups varies from *c*. 30 to 80% between years; Covas *et al*. 2006). Helpers are predominantly males (75% in a previous study; Doutrelant *et al*. 2004) and mainly offspring of one or both breeders (93%), although a small number of unrelated birds can also help (Covas *et al*. 2006).

### Field methods

The work was conducted at Benfontein Nature Reserve in the Northern Cape Province of South Africa (28°52′ S, 24°50′E), with the permission of the Northern Cape Department of Tourism, Environment and Conservation and the approval of the Ethics Committee of the University of Cape Town. The study area covers approximately 15 km^2^ of Kalahari sandveld, consisting of open savanna dominated by *Stipagrostis* grasses and the camelthorn tree, *Acacia erioloba*. The area is semi-arid, experiencing low and unpredictable rainfall (average 431 ± 127 mm per year; Weather Bureau, Pretoria). The study area contains about 30 sociable weaver colonies. This study was conducted at 23 of those colonies, although the number of colonies caught each year varied from 10 to 23. Colonies have been captured regularly since 1993, yielding a minimum age (i.e. the first time an individual was captured) for all individuals included in this study (*n* = 168). In addition, exact ages (based on young ringed at the nest or first caught in juvenile plumage) were known for 28 breeding birds (16% of the birds included here).

Since 1999, the resident birds at each colony were captured annually (except in 2007) before the onset of the breeding season by placing mist nets around the colony before dawn (i.e. when the birds are roosting within the nest structure) and then flushing the birds into the nets (Covas *et al*. 2002). Individuals were processed and released at the site of capture. All individuals were given a unique numbered aluminium ring and colour-ring combination.

We monitored breeding activity by inspecting all nest chambers in the study colonies every 3-4 days during 5 breeding seasons (1999–2000, 2000–2001, 2008–2009, 2010–2011 and 2011–2012). Nest chambers were individually marked with a numbered plastic tag. To identify the individuals feeding at a given chamber, and hence the number of helpers, we conducted a minimum of 1 h daily observations for at least three consecutive days (Covas *et al*. 2006; Doutrelant & Covas 2007). Observers were situated in a hide placed at 3–5 m from the colony. We obtained data on breeding group composition for 168 breeders (85 females and 83 males). Then, from 2000 to 2005 and from 2008 to 2013, we used capture–mark–recapture data to estimate survival. Birds captured in 2006 were not considered here because no birds breeding in 1999 and 2000 were recaptured after 2005.

**Fig. 1:**
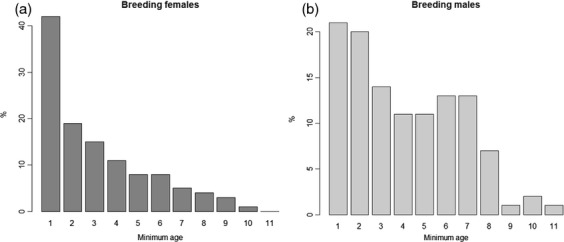
Histogram showing minimum age distributions for breeding females (a) and males (b).

The minimum age of the breeders varied from one to 11 years (Fig. 1). The exact age of some individuals (*n* = 28) was known if they were first ringed as nestlings or fledglings (in their first 4 months after fledging sociable weavers can be easily aged through the development of the black bib). The age of these 28 breeders (18 males and 10 females) varied from 2 to 10 years for males and from 1 to 5 for females.

To examine how representative minimum age was of real age, we investigated the likelihood that a newly captured bird (breeder or non-breeder) was a 1-year-old bird in 10 colonies monitored for the entire breeding seasons in 2010 to 2014. For those individuals of known sex captured for the first time as an adult at these colonies between 2011 and 2014 (*n* = 371 individuals), we found that 71% of the females and 81% of the males were chicks from the previous year (and thus definitely 1-year-old birds) and thus, respectively, 29% and 19% were immigrant birds (which may have been 1-year-old birds, but possibly older). Thus, minimum age is close to real age for a large proportion of birds.

Rainfall influences food availability and the duration and success of the breeding season in sociable weavers (Maclean 1973b; Dean & Milton 2001; Covas, du Plessis & Doutrelant 2008) and can thus influence survival (Altwegg *et al*. 2013). To control this factor, we obtained rainfall data from Kimberley Airport (28°48′ S, 24°46′ E; *c*. 10 km from the centre of the study site) and included it as a covariate in our analyses. For the present analyses, we used summer rainfall (from September to June), which coincided with the breeding season. Summer rainfall during the study period ranged from 251 to 807 mm.

### Molecular determination of the sex and identity of the parents

Since sociable weavers are sexually monomorphic, sex had to be determined through molecular techniques. The breeders' sex was determined by amplification of chromo-helicase-DNA-binding genes located on the W and Z sex chromosomes using the P2 and P8 universal primers (Griffiths *et al*. 1998).

To determine whether a bird seen at a nest was a breeder or helper, we used microsatellite markers to determine parentage. For 1999–2000 and 2000–2001, we used the results of parentage analyses presented in Covas *et al*. (2006). For 2008–2009, 2010–2011 and 2011–2012, we determined parentage based on 17 microsatellites markers. Blood samples were taken from the brachial vein for all adults captured and all offspring included in the study. Total genomic DNA was extracted using a modified ammonium acetate precipitation method. The DNA content of the extractions was quantified using a Nanodrop ND8000, and then, each sample was genotyped using 17 microsatellite loci for genotyping (PS1-GCSW15, GCSW47, INDIGO40, TG22-001, PS2-GCSW35, INDIGO41, Ppi2-Gga, TG01-148, WBSW9, PS3-GCSW13, INDIGO29, CAM1, CAM15, PS4-Ase18, GCSW31, GCSW57, TG07-022 Martinez *et al*. 1999; McRae & Amos 1999; Richardson *et al*. 2000; Sefc, Payne & Sorenson 2001; McRae *et al*. 2005; Dawson *et al*. 2010, 2013). These were grouped into four primer sets using a Qiagen Mastermix kit.

PCR product was sequenced using an ABI3730 capillary sequencer using the GeneScan™ 500 ROX™ Size Standard (Applied Biosystems), and results were analysed using genemapper v3.7 software (Applied Biosystems, Foster City, CA, USA). All of the scores were checked manually and adjusted wherever the genotype call was deemed to be in error.

The program cervus v3.0.3 (Tristan Marshal, Field Genetics Ltd, London, UK) was used to quantify the number of alleles and the observed and expected heterozygosity and to check for null alleles. The program genepop (http://genepop.curtin.edu.au) was used to test each locus for conformity to Hardy–Weinberg equilibrium (HWE) and to check for linkage disequilibrium (LD) between loci.

The program colony v2.0.3.5 (Jones & Wang 2010) was used to assign each chick a most likely mother and father through a likelihood approach. We used the genotypes of 181 offspring and used all genotyped male and female adult birds in the whole study population as parent candidates (529 females and 561 males). The proportion of candidate parents sampled was set at 75% to simulate the chance that an unknown individual might be the parent. A rate of 1% marker typing error was set. Fathers and mothers were assigned when their output parentage probability was given as 1. As previously reported (Covas *et al*. 2006), we did not find any evidence of extra-pair or extra-group paternity in this study (100% of identified incubating males and females were found to be the parents of the whole brood, and 100% of the 83 genetically assigned fathers were seen feeding the nestlings).

### Statistical methods

We tested for differences in survival according to the number of helpers using multistate multi-event capture–recapture (CR) models with state uncertainty (Pradel 2005), using the software e-surge v1.8.9 (Choquet, Rouan & Pradel 2009) and following a maximum-likelihood procedure from the capture–recapture histories of the birds. In this type of analysis, the probability of encountering a marked individual is the product of four probabilities: the survival probability, the probability of changing status (here the number of helpers), the probability of recapture and the ability to attribute a state (number of helpers) to an individual (state uncertainty). Individual capture histories were built for 168 birds with known number of helpers (from 0 to 4) for at least one reproductive event. Each breeder was assumed to have one of the 6 possible states every year (from zero to four helpers and dead). In all models, we allowed the probability of transition in the number of helpers from the year t to the year t + 1 to vary depending on their initial number of helpers during year t, as in McGowan, Hatchwell & Woodburn (2003). To simulate state uncertainty, the capture histories were constituted by seven possible events: from seen breeding without helpers to seen breeding with four helpers, not encountered, and a seventh event corresponding to unknown status. The certainty in assigning the number of helpers (i.e. the proportion of birds caught for which we subsequently identified the breeding group size) was set up to vary between the different monitored breeding seasons but fixed at 0 for the years when the breeding group composition was not studied.

By analysing individual capture histories, it is possible to distinguish a probability of survival (Φ) from a recapture probability (*P*), which is not the case when simply studying return rates (Gimenez *et al*. 2008). The simplest model Φ(.)+*P*(.), where both survival and recapture are constant, returned an overall survival and recapture probabilities of 0·72 ± 0·02 and 0·78 ± 0·03, respectively. We first verified that our data set met the expectations of the Cormack–Jolly–Seber (CJS) assumptions (no trap-dependence and no transient effect), using program U-Care (Choquet *et al*. 2009). The test of goodness-of-fit on CJS indicated that this model offered a satisfactory fit to the data set allowing the use of CMR statistics (goodness-of-fit test, global test, quadratic 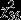 = 19·2472, *P* = 0·99).

In these analyses, we were mainly interested in the effect of the number of helpers on parental survival. However, a number of other factors could have affected survival and also had to be tested. To limit the number of parameters estimated simultaneously (Gregoire *et al*. 2004), we first tested the effect of year, minimum age, helpers and sex on both survival and recapture probability. We selected the best model, which here was Φ(.)+*P*(t+h), indicating that survival probability (Φ) was constant and the recapture probability (*P*) varied with time (t: i.e. between years) and negatively with the number of helpers (h).

We then tested the effects of several other variables of interest on the survival probability. These explanatory variables were as follows: the number of helpers, and the minimum age of the focal breeder (implemented in e-surge to increase every year as in Péron *et al*. 2010), the body mass of the focal breeder, and rainfall, all of which have previously been found to influence sociable weaver survival (Covas *et al*. 2002; Altwegg *et al*. 2013). In addition, we were interested in whether the effect of helper number could interact with other factors. We included only interactions that were considered biologically relevant *a priori* (Burnham & Anderson 2002). Specifically, we tested whether the presence of helpers could have an effect only under low rainfall conditions (Covas, du Plessis & Doutrelant 2008) or affect only one of the sexes (see Introduction). We investigate possible correlations between explanatory variables and the number of helpers did not vary with the minimum age or body mass of the male or female breeders or with rainfall (glmm with Poisson distribution, random term ‘individual identity’; null model with lower AIC; no effects were found even when considering the presence of helpers as a binary variable and a binomial distribution).

We tested hypotheses by comparing different models using the Akaike information criterion corrected for sample size (AIC_c_). This method simultaneously optimizes the deviance explained and the number of parameters (Akaike 1998). The model with the lowest AIC_c_ is the best, whereas models that differ by ΔAIC_c_ < 2 are considered to have equivalent support (Burnham & Anderson 2002).

## Results

**Fig. 2:**
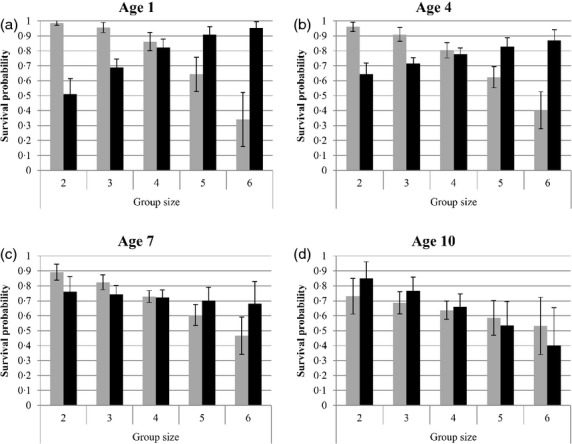
Predicted survival probability of breeding males (in grey) and females (in black) in relation to breeding group size and minimum age (ages 1, 3, 5 and 7 are shown). Values shown are parameter values from the model Φ(h*a*s)+ *P*(t+h) (Table 1).

One model shows an AICc that differs by more than 2 from all other models. This best supported model includes a triple interaction between age, sex and the number of helpers on breeders' survival probability (Table 1, Fig. 2). This model showed that the survival of young females (or recent immigrants, for which real age is unknown, but minimum age is low) increased with the number of helpers (varying from 50·9% without helpers to 95·4% with four helpers for females of minimum age 1, Fig. 2a). This positive effect of helpers on breeder survival diminished as females aged (Fig. 2b–d). By contrast, the survival of ‘young’ males strongly decreased with helpers' numbers (varying from 98·7% without helpers to 34·1% with four helpers for males of minimum age 1, Fig. 2a). This negative effect of helpers on male breeder survival persisted for all age categories (Fig. 2b–d).

**Table 1:** Modelling the survival probability (Φ) and recapture probability (*P*) in relation to the presence of helpers (h) and other covariates (s = sex, r = rainfall, a = minimum age, m = mass, t = time). The best model is in bold (ΔAIC_c_>2)

Model	AIC_c_	Δ AIC_c_	K	Deviance
φ(h^*^a^*^s) *P*(t+h)	1673·93	0	36	1595·19
φ(h^*^s+a^*^s) *P*(t+h)	1676·44	2·51	34	1602·45
φ(h^*^s+a^*^s+m) *P*(t+h)	1677·03	3·10	35	1600·66
φ(h^*^s+a) *P*(t+h)	1677·11	3·18	33	1605·47
φ(h^*^s) *P*(t+h)	1677·35	3·42	32	1608·06
φ(h^*^s+r) *P*(t+h)	1677·79	3·86	33	1606·15
φ(h^*^s+a+r) *P*(t+h)	1678·13	4·20	34	1604·13
φ(h^*^s+m) *P*(t+h)	1678·97	5·04	33	1607·33
φ(h^*^s+h^*^r) *P*(t+h)	1679·94	6·01	34	1605·94
φ(h^*^s+h^*^m) *P*(t+h)	1680·64	6·71	34	1606·65

The triple interaction is partly due to the important interaction between sex and the number of helper (present in all 10 best models, Table 1). In order to better understand which other effects were responsible for this triple interaction, we further investigated the effect of the interaction between minimum age and helper number on males and females separately. For females, the model including this interaction presented a lower AICc than the model including the simple effects of minimum age and helper number (ΔAICc = 1·68) In contrast, the model including the interaction was slightly higher for males (ΔAICc = 0·87) suggesting no interaction between age and helper number in males. Thus, these additional analyses indicate that the triple interaction between age, sex and helper number is due to an interaction between sex and helper number and an interaction between helper number and minimum age for females only.

## Discussion

The aim of our study was to test the hypothesis that sociable weaver helpers have a positive effect on breeders' survival and, moreover, that this effect is stronger for females than for males. Given that the cost of reproduction should be higher earlier in life, when breeders are less experienced, and the probability of breeding again is also higher earlier in life, we further expected the presence of helpers to be more beneficial for younger birds. Our results provide strong evidence of a positive effect of helpers on female survival early in life. Unexpectedly, however, we also found evidence of a negative effect of the number of helpers on breeding males' survival probability. These are correlative results and therefore may not reflect causality. Nonetheless, the strong association found is highly suggestive of antagonistic effects of helpers on female and male breeders' survival and that these effects vary with age, thereby revealing a complex effect of helpers on breeders' fitness.

### Female survival increases with the number of helpers: a consequence of load-lightening?

The effect of helpers on females' survival may be due to the reduced workload by breeding females in the presence of helpers. Like many cooperative breeders, females reduce the rate at which they provision nestlings when helped (Covas, du Plessis & Doutrelant 2008), but reduced investment by females in egg production in anticipation of being helped is another potentially important benefit. We showed in a previous study that sociable weaver egg mass decreased by, on average, 1·67% per additional helper (Paquet *et al*. 2013). Egg production is costly for birds (Monaghan & Nager 1997) and sociable weavers have protracted breeding seasons which may last 10 months, during which time females can lay up to 14 clutches, mostly to replace those predated by snakes (Paquet, Doutrelant & Covas; pers. obs.). The effect of helpers on the survival of breeding females may therefore arise from a reduction in their cost of reproduction. Similarly, in superb fairy-wrens *Malurus cyaneus*, in which females also produce lighter eggs in the presence of helpers (Russell *et al*. 2007), females but not males were also found to have a greater recapture rate in the presence of helpers (Cockburn *et al*. 2008).

Interestingly, we found that the positive effect of helpers on females' survival was only detectable for younger females (or those that immigrated recently into the study colonies, which are presumably young females since in our study colonies females usually disperse to breed when 1–3 years old). This is in agreement with a load-lightening strategy being more beneficial early in life when the potential number of future breeding attempts is higher. In addition, the positive effect of helpers on reproduction may be stronger for younger birds, as found for yearling female scrubwrens *Sericornis frontalis* (Magrath 2001); in sociable weavers, the effect of helpers on reproductive output is also greater under poor conditions (Covas, du Plessis & Doutrelant 2008). Further data are needed to study the direct relationship between egg mass, age and female survival, and hence to test the hypothesis that higher survival of young females in the presence of helpers is driven, at least in part, by energy saving during egg laying.

The increased survival of females in the presence of helpers could also be due to better maternal quality, if better quality females are more likely to be assisted by helpers. We attempted to guard against this possibility by testing for an effect of individual body mass on survival, finding no significant effect. However, body mass may be a poor proxy for individual quality, so this test does not allow us fully to distinguish these two non-mutually exclusive possibilities. Correlative studies on the effects of helpers on breeders' fitness, such as this one, are plagued by the possible confounding effects of better quality individuals having higher reproductive success, and hence more helpers, in the following year (Cockburn 1998). However, in our study, young females aged 1–2 years (when the positive effect of helpers on survival is stronger) are usually breeding for the first time, which argues against the possibility that the relationship between helper presence and female survival arises from higher quality females having had higher reproductive success in the previous year. Nonetheless, it is possible that the highest quality females have access to widowed males that have previously bred successfully and have offspring that can act as a helper workforce for the current brood. A longer longitudinal study of breeding group composition may provide more conclusive answers to these questions, allowing comparisons of the productivity of the same pairs in years with and without helpers (Cockburn *et al*. 2008). Nonetheless, the interaction that we detected between breeder age and the number of helpers, showing a decrease in the benefits to breeding females of being assisted by helpers, suggests that there is a real effect of helpers on female survival that goes beyond any possible correlation between female quality and the possibility of breeding with helpers.

### Why does male survival decrease with the number of helpers?

The strong negative effect of the number of helpers on the survival of breeding males was unexpected. This result was particularly surprising because although breeding male sociable weavers seem to feed the young at higher rates than either females or helpers (Doutrelant & Covas 2007), both sexes reduce their provisioning rates in the presence of helpers (Covas, du Plessis & Doutrelant 2008), such that we might expect survival benefits of helpers for both males and females. We suggest that there are at least five possible explanations for this odd finding: a confounding age effect, intragroup competition to mate with the breeding female, intragroup competition for breeding position, extra-group competition and an increase in dominance interactions. We now consider each in more detail.

First, the strong negative effect of helpers on male survival could have been explained if age was positively correlated with the presence of helpers. However, this was not the case for both sexes (see Methods). In addition, we included minimum age in our model.

Secondly, we might speculate that a potential sex-specific cost of helper presence might have arisen from competition between fathers and helpers, notably for reproduction. In superb fairy-wrens, for instance, the absence of helper effects on males' survival was attributed to the costs of the higher rates of extra-pair paternity that are associated with greater numbers of helpers (Mulder *et al*. 1994; Dunn & Cockburn 1999; Cockburn *et al*. 2008). However, this is an unlikely mechanism in sociable weavers since there is no evidence of extra-pair paternity in our population (Covas *et al*. 2006).

Thirdly, competition with helpers for reproduction may exist even in the absence of EPP. For example, in the only other reported case of a male specific negative effect of helpers' number on survival – in Alpine Marmots *Marmota marmota* – males compete with helpers for reproductive tenure rather than paternity (Allainé & Theuriau 2004; Lardy *et al*. 2012). In sociable weavers, a non-negligible number of male helpers (34%) were found to be unrelated to the breeding female and thus may indeed compete with the breeding male for access to that female in subsequent years (Covas *et al*. 2006). The probability that helpers compete with the breeding male to take over the breeding position inside a group still needs to be further investigated in this species, but we found no evidence of divorces from 1 year to the next in the present data set, suggesting that even if such costly competition exists, it is effectively blocked by the breeding male.

Fourthly, the effect of helpers on male survival may be the result of a confounding effect of competition outside the breeding group in the colonies. The number of helpers in our population fluctuates greatly in relation to productivity in the previous breeding season and current breeding conditions (Covas *et al*. 2004; Covas, du Plessis & Doutrelant 2008). In years following very productive breeding seasons, both breeding group size and colony size increase. Indeed, breeding group size and change in colony size were positively related in our data base (lme; model including an effect of helpers' number on colony size variation better than the null model by 15 AIC). Under these conditions, we might expect an increase in competition for resources and breeding chambers, leading to more aggressive interactions among males, who are more likely to engage in dominance interactions than females (Rat *et al*. 2014), resulting in reduced male survival. To investigate this possibility, we tested for any effect of change in colony size on male or female return rate, but found none (glmm with binomial distribution; null model with lower AIC). The presence of helpers may also be associated with costs of non-sexual competition inside the group. For example, Seychelles warblers (*Acrocephalus sechellensis*) had a lower survival probability when in larger groups, which may be the consequence of competition for resources (Brouwer *et al*. 2006). In sociable weavers, this mechanism would predict an interaction between the number of helpers and rainfall, given that competition for food is more likely during dry years, but we found no such interaction and the number of helpers did not depend on annual rainfall in our data set, suggesting no confounding effect of environmental conditions on helper number.

## Conclusion

Even though the mechanism underlying the negative impact of helpers on male breeders' survival remains to be investigated, our finding that both the costs for males and the benefits for females decrease with age is crucial to understand overall costs and benefits of helping across an individual's life span. Indeed, males start to breed later in life than females and hence the minimum age of males included in this analysis is higher than that of females (Wilcoxon test: W = 5897, *P* = 0·002, Fig. 1). This suggests that most females benefit from the presence of helpers in terms of their own survival. In addition, the survival costs and benefits that we have described here represent just one effect of helpers on breeders' fitness, and an understanding of the overall effect of cooperative breeding for the inclusive fitness of helpers and breeders requires further investigation of other potential direct and indirect fitness benefits.

In summary, we have shown antagonistic effects of helper presence on breeding male and female survival. There is a strong positive effect of helper number on breeding female survival early in life, but also a negative effect on breeding male survival. Thus, selection on each sex for breeding in cooperative groups differs. Although further study is needed to obtain a better understanding of the mechanisms underlying the effects reported here, this differential effect of helpers' presence on males and female survival has important consequences for the fitness of each sex and hence for our understanding of the evolution of helping behaviour. Sex- and age-specific effects of helper presence in cooperatively breeding species remain poorly studied; yet, survival is a key component of fitness, particularly in long-lived species such as most cooperative breeders. We hope that the present results will encourage more detailed studies of helpers' effects on breeders' survival in other species and, ultimately, how this will contribute to lifetime fitness.

## Data accessibility

Data are available from Dryad digital repository http://dx.doi.org/10.5061/dryad.mk44c (Paquet *et al*. 2015).
